# Security and Privacy of Cloud- and IoT-Based Medical Image Diagnosis Using Fuzzy Convolutional Neural Network

**DOI:** 10.1155/2021/6615411

**Published:** 2021-03-18

**Authors:** J. Deepika, C. Rajan, T. Senthil

**Affiliations:** ^1^Department of Information Technology, Bannari Amman Institute of Technology, Sathyamangalam, Erode, Tamilnadu, India; ^2^Department of Information Technology, K S Rangasamy College of Technology, Tiruchengode, Namakkal, Tamilnadu, India; ^3^Department of Electronics and Communication Engineering, Bannari Amman Institute of Technology, Sathyamangalam, Erode, Tamilnadu, India

## Abstract

In recent times, security in cloud computing has become a significant part in healthcare services specifically in medical data storage and disease prediction. A large volume of data are produced in the healthcare environment day by day due to the development in the medical devices. Thus, cloud computing technology is utilised for storing, processing, and handling these large volumes of data in a highly secured manner from various attacks. This paper focuses on disease classification by utilising image processing with secured cloud computing environment using an extended zigzag image encryption scheme possessing a greater tolerance to different data attacks. Secondly, a fuzzy convolutional neural network (FCNN) algorithm is proposed for effective classification of images. The decrypted images are used for classification of cancer levels with different layers of training. After classification, the results are transferred to the concern doctors and patients for further treatment process. Here, the experimental process is carried out by utilising the standard dataset. The results from the experiment concluded that the proposed algorithm shows better performance than the other existing algorithms and can be effectively utilised for the medical image diagnosis.

## 1. Introduction

The development in the medical field and the advancement of medical devices such as magnetic resonance imaging (MRI) and computed topography (CT) produces huge data on daily basis, and the data collected from these devices are high dimensional and rich in variables [1]. Thus, the medical image databases and their dimensionality are increasing tremendously. Due to this increase in medical databases, it is difficult to handle the file system with increasing data volume. Thus, handling of medical data turns into a biggest concern for healthcare service providers [2]. Hence, cloud computing was utilised in the medical field for storing and computing the medical data since the medical image cloud is easy to handle. Cloud computing offers flexible and scalable computing resources from the distant locations, and they are accessible depending on the necessity of the user [3]. It is also efficient for delivering computing resources in the high end-computing environment [4]. For cloud computing, the users do not need their own storage and servers at their computers because the cloud platform is utilised for data storage. Hence, the users will be free from having large storage and huge number of servers in their computers [5]. Therefore, computing and storage of long-term medical image records in the cloud are efficient for solving many problems in the medical field [6]. So cloud computing is widely used in the medical field for storing, computing, and sharing the patients' medical records. In cloud computing, the hospital only needs to collect the information about the patients from the files and transfer the data to the cloud for storage.

Moreover, Internet of Things (IoT) is also extended in cloud computing for developing new facilities and applications in the healthcare process [7]. IoT is defined as the network of things or objects such as sensors, software, and electronic devices interconnected with each other for exchanging data with the operator, manufacturer, or other connected devices to attain greater values and services. Also, IoT  offers advanced connectivity among the systems, services, and devices which includes various domains, protocols, and applications. IoT and cloud computing are profited equally while combining the technologies. The IoT technology always supports the cloud for enhancing the performance such as computational ability, energy, storage, and high resource utilization. Also, it favours the cloud to provide many new services through a distributed and active approach.

While storing medical data in the cloud platform, it is necessary to safeguard the information so that the cloud cannot learn anything about the data. Thus, securing the medical images in cloud platform is necessary. Generally, medical images are highly sensitive to modifications, and thus, any alterations in their contents can cause errors in medical diagnosis [8]. Hence, it is also important to maintain the sensitive contents of medical images during a reconstruction phase. Thus, an encryption algorithm is required for increasing the security and privacy of data which secures the medical data without leaking any sensitive information. Many encryption algorithms are proposed based on chaotic systems like 1D and 2D chaotic systems, but these chaotic systems with low dimensions only contain simple orbits and lesser parameters. Thus, the initial values and parameters can be easily estimated from the image [9]. To minimise these limitations, chaotic system based on backpropagation (BP) neural network is utilised for the image encryption process which effectively secures the medical data in the cloud environment.

For medical image analysis and disease diagnosis, machine learning technique is extensively used in the medical field [10]. It has many functions such as pattern recognition, disease prediction, fraud detection, and image segmentation [11]. However, the conventional machine learning techniques are not adequate for the large medical databases. Thus, high performance computing was employed in machine learning data for accurate and efficient diagnosis of big medical data. A division of machine learning process is the deep learning which depends on learning data illustrations for feature classification, and it utilises supervised and unsupervised machine learning methods. It is an advanced technique which has the ability to discriminate features of data without human intervention. It has an ability to consequentially produce robust and representative features layer by layer in neural networks [12]. Data used for deep learning are obtained from different sources with precise data types, and it is significant to develop suitable models for handling data analysis. The typical modelling of data analysis includes clustering model, classification model, neural network model, and other efficient models. Neural network is considered as a vital element in deep learning, which replicates biological systems for communication node distribution and information processing. Deep learning process consists of various types such as recurrent neural network (RNN), restricted Boltzmann machine (RBM), autoencoder (AE), multilayer perceptron (MLP), and convolutional neural network (CNN) [13–16].

The storage, handling, and processing are challenging for large sized medical images, and thus, high performance processors are required for medical image processing [17]. Hence, CNN is utilised in the medical field for processing large volume of medical data. CNN is a feed-forward neural network which helps in the modelling of sequential data. It also helps to easily predict and classify the disease and aids in decision-making during disease diagnosis by utilising various approaches. Thus, it assists the clinicians to detect and characterize the important features in large image series. Convolutional neural networks are utilised for different computer applications such as superresolution, medical image classification, and sematic segmentation [18]. They are also used for detecting the objects from satellite image and for implementing many real-time applications [19]. Convolutional neural network effectively learns the local and global features from the image dataset, so it is widely employed in the image classification process. CNN utilises the supervised learning techniques, and thus, it gives better classification results than the unsupervised techniques like RBM neural networks [20].

Convolutional neural networks enable accurate and robust big data feature learning. They utilise many samples for extracting meaningful information from data. Yet, the data may be ambiguous because of inadequate information or complexity of the data source which is known as data ambiguity. Instead, the fuzzy logic is the effective tool for modelling human thinking and perception. It offers the mathematical model for processing ambiguous data by executing numerical computations using linguistic labels and fuzzy set degrees of membership, but the fuzzy systems lack learning ability and the fuzzy rules are determined by the human experts. Thus, the fuzzy system is combined with neural networks so that the fuzzy rules can be derived from a large source of training data by automatically learning the membership functions. Hence, fuzzy convolutional neural network is proposed by integrating fuzzy logic and convolutional neural networks. It utilises the advantages of both fuzzy logic and CNN for accurate and robust classification of data.

This paper is formulated as follows: the literature survey related to the article is explained in Section 2. The architecture of the proposed system is described in Section 3. In Section 4, the proposed methodology regarding the secured cloud storage and medical image processing is explained.  Section 5 gives the experimental results for the proposed system. Finally, Section 6 illustrates the conclusion and future works.

## 2. Literature Survey

Due to the momentary advancement in the healthcare services, there is a rapid growth in medical data. The healthcare services in medical field involve diagnosing the diseases, treatment, injury, disease prevention, and treating other mental or physical injuries in patients. The quality of these healthcare sectors lies in the problem detection efficiency and allocation of medical resources which require application and collection and management of medical data [21]. Previously, many organisations used manual records about the patients organized in the form of reports [22], but for large number of datasets, a modern technology is required in data analysis and management. Hence, cloud computing technology and IoT have been introduced in the medical field for effectively handling, storing, processing, and transferring the medical information of patients. Cloud computing is a smooth and dynamic process which offers a reasonable strategy for handling medical data [23], whereas Internet of Things provides interconnectivity among the devices, applications, data, objects, and sensors which highly influence the data transmission process in medical field. Thus, IoT provides continuous communication and interaction between the sensors and devices in the cloud computing environment. Now, many organisations have initiated to utilise the IoT and cloud computing technology due to its convenience in their operations [24]. Also, cloud computing and IoT are utilised by pharmaceutical and medical research organisations whose applications can have great impact for the welfare of society.

The developments in the medical technology made the storage of medical data in cloud become more convenient. The medical images contain much significant information about patients, and thus, the secure transmission of data and secure storage is important. Hence, for the security of the data, the encryption and the decryption algorithm has been utilised. The chaotic system is extensively applied in the encryption process as it provides better results for randomness and sensitivity of parameter and initial values [25]. Many investigations have been conducted by utilising chaotic system in encryption algorithms. Zhou et al. [26] suggested 1D chaotic system for the encryption process, and Cao et al. [27] offered 2D chaotic system for the encryption process. These are low-dimensional chaotic system with simple orbits and few parameters, and thus, the initial values and parameters can be estimated easily. Gupta and Silakari [28] suggested an image encryption algorithm by utilising 3D cat map. Then, Huang and Nien [29] analysed about the colour image encryption process by utilising 3D chaotic system. Also, Wu et al. [30] proposed the 6D hyperchaotic algorithm for colour image encryption. However, these algorithms have less safety factors including low differential attraction, larger correlation coefficients, and smaller key space. Thus, for improving the safety features of the image encryption algorithm, the backpropagation (BP) neural network- and chaotic system-based encryption algorithm was proposed for the image encryption process.

The progressions in the data storage capabilities, computational resources, hardware design, and safety procedures have increased the necessity of medical data. The medical data undergo processing for effective analysis of diseases using data processing techniques. The medical data processing includes classification, segmentation, and abnormality prediction by utilising the images produced from the medical devices. For large volume of medical data, an efficient data processing technique is required for collecting the valuable insights from datasets. Deep learning [31, 32] is a part of machine learning which has the ability to classify the data based on feature learning without manual interference. It is the widely applied machine learning technique [33] which can produce robust and representative features automatically [34, 35]. It is especially suitable for analysing large dataset and also utilised in speech analysis, computer vision, natural language processing (NLP), scene classification [36], and face recognition [37]. Many investigations have been conducted on image classification by utilising deep learning algorithms like convolutional neural networks and stacked autoencoder (SAE) [30].

Among the deep learning methods, the convolutional neural network provides better classification accuracy with most powerful architecture [38]. CNN is a deep learning process used for solving complex classification problems [12]. It improves the computational performance and precision rate for large datasets. Also, it automatically extracts the feature maps in association with training data. During the classification process, the model is pretrained with related and sufficient data through which the feature description of the model is assigned. Thus, the model begins with the pattern which is related to the task rather than starting from certain random values. Still, the learning process needs training dataset related to testing dataset because the CNN cannot perform efficiently without relevant data [39]. Thus, the convolutional neural networks are combined with fuzzy logic system to form fuzzy convolutional neural network for modelling nonlinear functions. This fuzzy convolutional neural network improves the effect of function approximation. Generally, the convolutional neural networks utilise the fully connected neural networks for analysing the information from feature maps. Here, the convolutional neural network is integrated with the fuzzy logic system instead of fully connected neural network to increase the ability to estimate ideal hypothesis.

## 3. System Architecture

**Figure 1:**
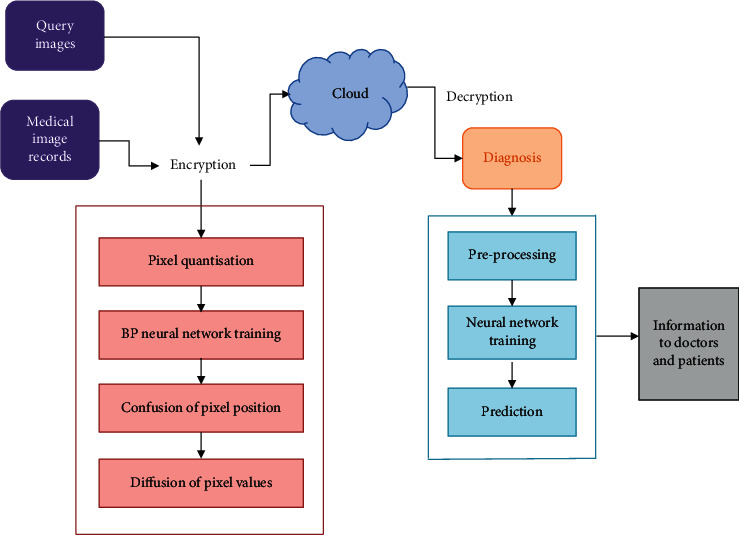
System architecture.

The conceptual structure of the proposed system comprises the following phases. At the initial phase, the necessary datasets such as query images and medical images are collected for image processing. The collected medical image datasets are then transferred to cloud subsystem using IoT and are stored into cloud database. Before transmission, these medical images undergo the encryption process to eliminate the security threats. This encryption process is carried out using BP neural network and chaotic system. After encryption, the medical will be transferred to the cloud platform where disease diagnosis takes place. The diagnosing process in the medical images is carried out using the proposed algorithm called fuzzy convolutional neural network algorithm which classifies the processed images into normal and disease affected. The classification results from the cloud are transferred to the doctors or experts in context of person's health by IoT. The architecture of the proposed system is displayed in Figure 1.

## 4. Proposed Methodology

### 4.1. Secured Cloud Data Storage and Transmission Using IoT

**Figure 2:**
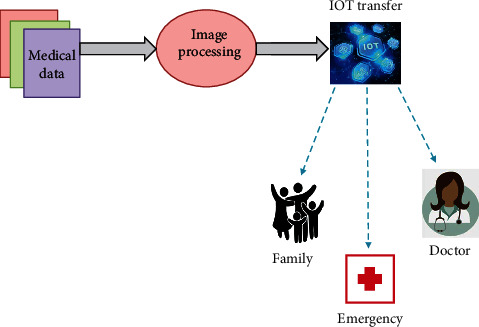
IoT-based medical image transmission.

The medical images are generally distributed to various organisations for accurate analysis of diseases. Thus, medical data sharing is necessary for improving the quality of healthcare services. The connectivity made through the Internet by IoT devices and the development in the cloud computing technologies made the users to utilise accessible and distributed cloud computing platforms. Here, the medical images are stored in the cloud where the image classification for disease diagnosis takes place. It classifies the images into normal and abnormal images, and the results are then transferred to the doctors and patients using IoT devices. IoT-based medical image transmission is given in Figure 2.

In cloud computing, the medical datasets are stored in the third party service provider which concerns the privacy of the user. Due to this privacy-related problems, the application of cloud sharing platforms is limited in the medical field. For secured data storage on cloud database, BP neural network- and chaotic system-based encryption algorithm is proposed in this system.

#### 4.1.1. Medical Image Encryption

The reason for implementing an encryption-decryption system is privacy. As information travels over the Internet, it is necessary to examine the access from unauthorized organisations or individuals. Hence, the data are encrypted to reduce data loss and theft. Encryption is the process in which the information is converted into secret code for concealing the actual information. The encryption and decryption processes are collectively called as cryptography. The algorithm utilised for encoding and decoding data is known as ciphers or encryption algorithms, while the unencrypted data are called plaintext and the encrypted data are known as ciphertext. The encryption process converts the plaintext into alternate form called ciphertext. The variable known as a key is required for decrypting the encrypted images. An authorized recipient can easily decrypt the message with the key provided by the originator to recipients but not to unauthorized users. Encryption is commonly used to protect data in transit and data at rest. Encryption has been a longstanding way for sensitive information to be protected. In modern times, encryption is used to protect data stored on computers and storage devices, as well as data in transit over networks. The time and difficulty in guessing the information is what makes encryption such a valuable security tool.

*(1) Preprocessing.* For preprocessing, the input image of size *P* × *Q* is used. The image size *P* × *Q* is disintegrated into *H* subimage blocks, and each block is given as *m* × *m*. For accelerating the convergence of network training, it is necessary to normalise the generated subimage blocks. Here, mean distribution pretreatment is used for normalising the grayscale image range which is given as [*X*_max_, *X*_min_], and the transformation domain is given as [*Y*_max_ − *Y*_min_]. The pixel value for processing is assumed as *X*_value_ and *Y*_value_ which is obtained as given in the following equation:

(1) $$\begin{array}{c} Y_{\text{value}}=\;\frac{\left(Y_{\max}-Y_{\min}\right)\left(X_{\text{value}}-X_{\min}\right)}{X_{\max}-X_{\min}}+X_{\min}. \end{array}$$

By equation (1), the pixel values of the plain image are distributed from [0, 1] through which the preprocessing of training samples takes place.

*(2) Image Compression.* After preprocessing, image compression takes place in the medical images by employing the BP neural network. During compression, the image data samples are given as the input. The number of hidden nodes in the network is denoted as *u*_*t*_, and the number of nodes in input layer is denoted as *u*_*i*_. The compression rate of BP neural network is denoted as *t*, and the association among them is given as follows:

(2) $$\begin{array}{c} t\;=\frac{u_{i}}{u_{t}}. \end{array}$$

During neural network training, the network coupling weight remains unaltered during the compression process. Thus, the newff function is employed for generating a trainable feed-forward network. The compression image is obtained by utilising newff function for training. The transfer functions are calculated as follows:

(3) $$\begin{array}{c} \log\text{sig}\left(n\right)=\frac{1}{1+e^{-n}}, \end{array}$$

(4) $$\begin{array}{c} \tan\text{sig}\left(n\right)=\;\frac{2}{1+e^{-2n}}+\;1. \end{array}$$

**Figure 3:**
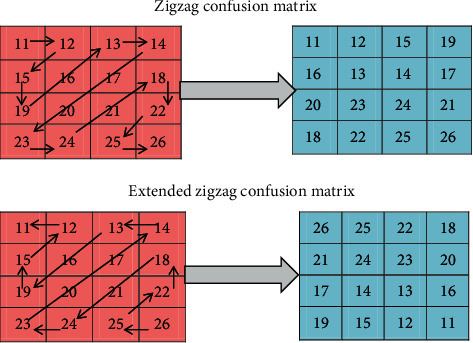
Zigzag confusion matrix and extended zigzag confusion for nonsquare matrix.

*(3) Image Scrambling Using Extended Zigzag Confusion.* The extended zigzag confusion algorithm is utilised for image encryption which scrambles the BP neural network trained image data. It is an extended form of zigzag confusion which is designed for eliminating the drawbacks in zigzag confusion. The scrambling process in zigzag confusion begins with the upper left corner of the matrix. During zigzag confusion, some values in particular positions are not altered even after scrambling for numerous times, and thus, it can be cracked easily. Also, the zigzag transformation is only suitable for square matrix and not suitable for nonsquare matrix. Hence, extended zigzag confusion is used which overcomes the limitations in regular zigzag confusion. In extended zigzag confusion, the scrambling process can begin from any of the four corners which are chosen by the random number created in the chaotic system. The extended zigzag confusion is also suitable for nonsquare matrix.  Figure 3 shows the zigzag confusion for square matrix and the extended zigzag confusion for nonsquare matrix.

For extended zigzag confusion, set the initial values and parameters of fractional-order chaotic system and then iterate the chaotic system *(m* *+* *P* *×* *Q)* times. For improving the sensitivity of initial values and parameters in chaotic system, the initial *m* values are discarded and three chaotic series are obtained. By combining these three series, two pseudorandom series *R*_1_ and *R*_2_ are formed. These series *R*_1_ and *R*_2_ are utilised for diffusing pixel values. Equations (5) and (6) give the xor diffusion algorithm.

Positive algorithm:

(5) $$\begin{array}{c} E_{i}=E_{i-1}⊕R_{i}⊕Q_{i}. \end{array}$$

Reverse algorithm:

(6) $$\begin{array}{c} E_{i}=E_{i+1}⊕R_{i}⊕Q_{i}, \end{array}$$

where *E* is denoted as ciphertext, which is a one-dimensional vector; *R* is denoted as the password vector; and *Q* is denoted as an expanded form of confusion image. Based on equations (5) and (6), the ciphertext vector *E* is formed which is the recovered pixel matrix. From the above equations, the encrypted image is attained.

#### 4.1.2. Medical Image Decryption

Generally, the data are encrypted to make it difficult for someone to steal the information. The conversion of encrypted data into its original form is called decryption. It is generally a reverse process of encryption which recovers the original image from the encrypted image. It transforms the data that has been rendered unreadable through encryption back to its unencrypted form. Thus, it decodes the encrypted information so that an authorized user can only decrypt the data because decryption requires a secret key or password. If a decryption passcode or key is not available, special software may be needed to decrypt the data using algorithms to crack the decryption and make the data readable. In decryption, the system extracts and converts the corrupted data and transforms it to texts and images that are easily understandable not only by the reader but also by the system. Decryption may be accomplished manually or automatically. It may also be performed with a set of keys or passwords. Decryption is taking encoded or encrypted text or other data and converting it back into text user or the computer can read and understand.

The decryption algorithm comprises inversing the pixel value diffusion, inversing the pixel position confusion, and reconstructing the BP neural network algorithm. For the decryption process, the encrypted image is given as input. Then, the inverse vectors S_1_ and S_2_ are obtained, and the inverse diffusion algorithms are expressed in equations (7) and (8).

Positive inverse algorithm:

(7) $$\begin{array}{c} P_{i}=C_{i-1}⊕C_{i}⊕S_{i}. \end{array}$$

Reverse inverse algorithm:

(8) $$\begin{array}{c} P_{i}=C_{i+1}⊕C_{i}⊕S_{i}. \end{array}$$

Then, using the extended inverse zigzag algorithm, the scrambled pixels are recovered. The pixel values quantization and the pixel blocks are retrieved, and the vectors also retrieved into subimage blocks. Finally, the decrypted image is formed by combining every subimage.  Algorithm 1 illustrates the encryption and decryption processes in medical images.

### 4.2. Medical Image Processing

Medical image processing plays a major role in the diagnosis of diseases and makes the treatment process more efficient. The medical image processing in this system is carried out using the fuzzy convolutional neural network. The medical images are generally collected as high dimensional type. Due to this high dimensional data, the functioning of the learning process in the fuzzy CNN can be weakened. Hence, before the implementation of fuzzy CNN, there is a requirement for dimensionality reduction and segmentation for improving classification performance.

#### 4.2.1. Improved Principal Component Analysis (IPCA)

Dimensionality reduction is very efficient for processing high dimensional images. The principal component analysis (PCA) is widely utilised for decreasing the dimensions of the original data. It utilises the linear transformation to create a simplified dataset while preserving the important features of original dataset. During principal component analysis, the output variables of eigenvectors are exposed through a variance and it has a huge impact in the analysis. This drawback in the analysis is improved using improved principal component analysis (IPCA). The IPCA requires lesser training time, and it experiences a smooth dimension reduction process.

*(1) Covariance Matrix Formation.* In covariance matrix formation, *X*_*n*_ is assumed as training vector and it is given as *X*_*n*_=[*x*_1_, *x*_2_,…,*x*_*n*_]^*T*^. The method for improved principal component analysis is illustrated as follows:

(9) $$\begin{array}{c} x_{sv}^{'}=\frac{x_{sv}\;}{1/n\sum_{s=1}^{n}x_{sv}},\;\;\left(s=1,2,\ldots ,n\text{ and }v=1,\ldots ,m\right), \end{array}$$

where the sample size is denoted as *n,* dimension number is denoted as  *m*, and the normalization value of *x*_*sv*_ is expressed as *x*_*sv*_′. In this process, there is no loss in information and the correlation matrix does not have any difference. The covariance *C*_*o*_ matrix is formed as follows:

(10) $$\begin{array}{c} C_{o}=\frac{1}{n}\;\sum_{1=1}^{n}\left[\left(x_{us}-\bar{x_{s}}\right)-\left(x_{uv}-\bar{x_{1}}\right)\right]. \end{array}$$

*(2) Calculation of Eigenvalue.* For decomposing the covariance matrix *C*_*o*_, the eigenvalue is calculated. It is expressed as

(11) $$\begin{array}{c} C_{o}=\sum_{y=1}^{N}\mu_{y}E_{y}E_{y}^{T}\;=E\Delta E^{T}, \end{array}$$

where the eigenvalue is denoted as *μ*_*y*_ and the rank of matrix is considered as *y*. The subeigen values of the vector in the orthogonal basis of [*E*_1_, *E*_2_,…, *E*_*y*_] is given as *E*_*y*_. The diagonal of matrix (*μ*_1_, *μ*_2_,…, *μ*_*y*_) is given as Δ, and we have *μ*_1_ > *μ*_2_ > ,…, *μ*_*y*_.

*(3) Principal Components Calculation.* After setting up the covariance matrix *C*_*o*_ and normalization, the original vector *M*_*n*_ is shifted to the uncorrelated vector *z*_*n*_, which is illustrated as

(12) $$\begin{array}{c} z_{n}=E^{T}M_{n}=\left[\begin{array}{ccc} \begin{array}{cc} e_{11} & e_{12}\\ e_{21} & e_{22} \end{array} & \cdots & \begin{array}{c} e_{1m}\\ e_{2m} \end{array}\\ \vdots & \ddots & \vdots \\ \begin{array}{cc} e_{m1} & e_{m2} \end{array} & \cdots & e_{mm} \end{array}\right]. \end{array}$$

Cumulative contribution rate *α*_*n*_ consists of information proportion of the initial *n* principal components. Also, the threshold value of *α*_*n*_ is taken as 85% for collecting necessary information. The contribution rate *α*_*s*_ and cumulated contribution rate *α*_*n*_ are determined from the following equations:

(13) $$\begin{array}{c} \alpha_{s}=\;\left(\frac{\mu_{s}}{\sum_{u=1}^{m}\mu_{s}}\right)\times 100,\;\left(k=1,2,\ldots ,m\right), \end{array}$$

(14) $$\begin{array}{c} \alpha_{n}=\sum_{k=1}^{n}\frac{\mu_{k}}{\sum_{k=1}^{m}\mu_{k}}>85\%,\;\left(u=1,2,\ldots ,q\right). \end{array}$$

After estimating the accurate value of *n*, the principal component of *P*_*s*_ sample is determined as follows:

(15) $$\begin{array}{c} P_{s}=\left[\begin{array}{c} P_{s1}\\ \begin{array}{c} P_{s2}\\ \vdots \end{array}\\ P_{sn} \end{array}\right]=\left[\begin{array}{cccc} \delta_{11} & \delta_{21} & \cdots & \delta_{q1}\\ \delta_{12} & \delta_{22} & \vdots & \delta_{q2}\\ \vdots & \vdots & \ddots & \vdots \\ \delta_{1n} & \delta_{2n} & \cdots & \delta_{qn} \end{array}\right]\;x_{k}^{T}, \end{array}$$

where (*u*=1,2,…, *q*).

#### 4.2.2. Image Segmentation Based on K-Means Clustering

Image segmentation is utilised efficiently in many medical imaging practices such as recognising the tumour in brain and determining the size of the tumour and its response to the treatment. Having good segmentation process will help the clinicians and patients as they present necessary information for 3D visualization, surgical planning, and disease detection. K-means clustering is the widely utilised method for image segmentation. It is an unsupervised learning algorithm which has relatively low computational complexity. Also, it is the simplest clustering method which divides a set of data into precise number of clusters. Generally, K-means algorithm identifies the number of clusters (K) in the particular regions of images, and thus, it is suitable for medical image segmentation [40].

K-means clustering is the data partitioning algorithm, where *n* observations are iteratively assigned to exactly one of the *k* clusters defined by centroids, where *k* is selected before the initiation of the algorithm. Here,  *C*={*c*_1_, *c*_2_,…, *c*_*k*_} is given as the set of *m* cluster centres and *Y*={*y*_1_, *y*_2_, *y*_3_,…, *y*_*m*_} is given as the set of  *n* data points. These *n* data points will be clustered into *m* cluster centres. The objective function of K-means clustering algorithm is given as

(16) $$\begin{array}{c} D=\sum_{i=1}^{m}\sum_{j=1}^{n}y_{i}-c_{j}^{2}\;, \end{array}$$

where *y*_*i*_ is the *i*^th^ data point and *c*_*j*_ is the *j*^th^ cluster centre. By minimising the objective function, the optimal centres can be attained. The initial cluster centre greatly influences the K-means clustering results. Thus, the cluster centres must be selected delicately.  Algorithm 2 illustrates the process of K-means clustering algorithm.

#### 4.2.3. Computational Complexity Analysis

The most relevant way to measure the complexity of an algorithm is in terms of how much time it takes to run (space, i.e., memory, can also be used as the basis for a complexity measure, but time is usually the more useful measure). For a complexity measure to reflect the intrinsic nature of the algorithm, it should summarize performance on all problem instances, and it should not reflect inconsequential implementation details.

Computational complexity is the method of measuring the complexity of the algorithm especially the running time of the algorithm. The space (memory) occupied by the algorithm can also be used for calculating the computational complexity, but time is generally more efficient than space. For reflecting the intrinsic nature of the algorithm, the complexity measure must summarize the performance on all problematic cases, and it must never reflect the insignificant implementation factors. The computational complexity of K-means clustering algorithm according to Algorithm 3 is illustrated.

  (i) Input: *n* denotes the number of clusters, Max_*t*_ denotes the maximum number of iterations, *y*_*i*,*j*_ denotes the data point allotted to *i*^th^ cluster centre, *N*_*i*,*j*_ denotes the total no. of data points allotted to *i*^th^ cluster centre, and *C*_*i*_ is the *i*^th^ cluster centre.
  (ii) Ensure: best cluster centre: *O*(Max_*t*_ × *n*).
  (iii) Initialization: *O*(1).
  (iv) Randomly select cluster centres *k*, *C*=*c*_1_, *c*_2_,…, *c*_*k*_: *O*(1).
  (v) Calculate the distance to each centre.
  (vi) Assign the data point to the cluster centre, whose distance from the data point is least of all cluster centres.
  (vii) Objective function evaluation, *C*_*i*_=(1/*N*_*i*,*j*_)∑_*j*=1_^*N*_*i*,*j*_^*y*_*i*,*j*_: *O*(*n*).
  (viii) Update the location of cluster centre: *O*(Max_*t*_ × *n*).
  (ix) Repeat steps 3 to 6 until the objective function *D* is minimised.
  (x) Iteration counterincrement: *O*(Max_*t*_).

#### 4.2.4. Fuzzy Convolutional Neural Network (FCNN)

**Figure 4:**
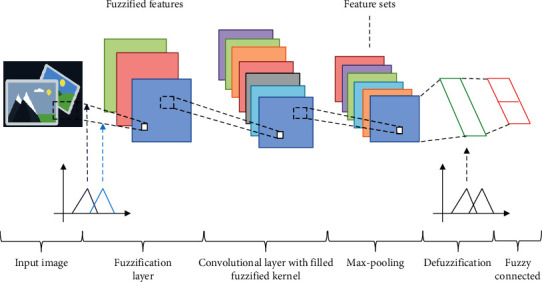
Structure of fuzzy convolutional neural network.

The image processing is complex in medical images because it has high resolution images. Thus, an effective classification algorithm like fuzzy convolutional neural network algorithm is utilised for medical image classification. The FCNN algorithm utilises both neural and fuzzy systems for the classification process. The information collected through CNN and fuzzy is merged together for creating the entire system for classification. In FCNN, fuzzy minimises the uncertainties and the neural network reduces the noises in the original data. The structure of FCNN is presented in Figure 4.

The structure of the proposed FCNN consists of convolutional layer and fully connected layer. The convolutional layer undergoes three stages such as convolution stage, nonlinearity stage, and pooling stage. At the initial stage, the input data undergo processing in the fuzzification layer for creating the fuzzy logic representation. Accordingly, the fuzzy representation is convoluted in the fuzzy convolutional stage which contains fuzzified convolution kernels. Fuzzy convolution is the min-max composition of fuzzified kernels which obtain higher level fuzzy features from its input spatial features. During the defuzzification layer, the crisp values are produced from the features obtained by pooling. In the final stage, a fully connected layer functions as an output classifier of FCNN.

To perform fuzzy inference, fuzzy rule is needed to be generated. For a multi-input method, every input is graded with each fuzzy set for its degree of membership. Next, inputs *x*_*i*_, *i*=1,2,…, *n* and outputs *y*_*j*_=1,2,…, *m* are assigned and the *k*^th^ fuzzy rule *R*^*k*^ is generated as follows:

(17) $$\begin{array}{c} R^{k}:\text{IF }x_{1}\text{ is }F_{1}^{k}\text{ and}\ldots x_{n}\text{ is }F_{n}^{k},\\ \text{THEN }y_{1}\text{ is }w_{1}^{k}\text{ and}\ldots y_{m}\text{ is }w_{m}^{k}, \end{array}$$

where *F*_*i*_^*k*^ is denoted as the fuzzy set with *i*^th^ input and *k*^th^ fuzzy rule. Based on membership functions, multiple linguistic labels are assigned for each element in input matrix. The input node membership to a certain fuzzy set is described as grade which is calculated by the fuzzy membership function. The fuzzy set ($\hat{X_{i}}$) is illustrated as

(18) $$\begin{array}{c} \hat{X_{i}}=\text{fuzz }\left(\frac{x_{ij}}{Mx_{ij}}\right), \end{array}$$

where the centre of input fuzzy membership function is denoted as *Mx* and the input matrix is denoted as *X*_*i*_.

The processing phases in the fuzzy convolutional layer include fuzzy convolutional phase, nonlinearity phase, and pooling phase. In the fuzzy convolutional phase, the fuzzy convolutional filter *Wt*_*μ*_ is applied to the original data, which is given as

(19) $$\begin{array}{c} x_{ij}=\sum_{a=0}^{m-1}\sum_{b=0}^{d-1}Wt_{\mu }x\left(i+a\right)\left(j+b\right). \end{array}$$

The fuzzy convolutional filter *Wt*_*μ*_ is calculated as follows with *Wt* as the original convolution filter:

(20) $$\begin{array}{c} Wt_{\mu }=\text{fuzz}\left(Wt\right). \end{array}$$

Equation (21) gives the nonlinear transformation of gained output from the fuzzy convolutional phase. The final phase in the fuzzy convolutional neural network is the max pooling phase. By this phase, the size of the input may be reduced in the next fuzzy convolution layer:

(21) $$\begin{array}{c} y_{ij}=f\left(x_{ij}\right). \end{array}$$

In the convolution phase, the activation function is denoted as *f*(.). The fully connected phase of fuzzy convolutional neural network works as a classifier with input features. By the process of defuzzification with centre of gravity, the crisp value *y*_*i*_ is calculated, which is illustrated as

(22) $$\begin{array}{c} y_{i}=\text{defuzzification}\left(x_{i}\right)=\frac{\sum M_{y}x_{i}}{\sum x_{i}}, \end{array}$$

where *M*_*y*_ denotes the centre of the defuzzification membership function. The output of the FCNN is represented as $\hat{z_{i}}$ which is given as

(23) $$\begin{array}{c} \hat{z_{i}}=W_{f}y_{i}. \end{array}$$

In the fully connected layer, the weight matrix is denoted as *W*_*f*_. For evaluating the output error, cross entropy is utilised. Equation (24) illustrates the estimation of cross entropy function:

(24) $$\begin{array}{c} \text{CE}=-\frac{1}{N}\overset{N}{\underset{n=1}{\sum }}\left[z_{n}\log\left(\hat{z_{n}}\right)+\left(1-z_{n}\right)\log\left(1-\hat{z_{n}}\right)\right), \end{array}$$

where the output of the classifier is given as $\hat{z}$, target is given as *z*, and the number of samples is denoted as *N.* The model parameters are trained using cross entropy loss function. Equation (25) gives the weight update:

(25) $$\begin{array}{c} W_{f}\left(k+1\right)=W_{f}\left(k\right)-\delta_{fc}\frac{\partial CE}{\partial W_{f}}. \end{array}$$

Equation (26) gives the updated defuzzification membership function, where the centres are denoted as *M*_*y*_(*k*), the learning rate is denoted as *δ*_*My*_:

(26) $$\begin{array}{c} M_{y}\left(k+1\right)=M_{y}\left(k\right)+\delta_{My}\nabla M_{y}. \end{array}$$

Equation (27) gives the updated fuzzification membership function with centre value as *M*_*z*_ and learning rate as *δ*_*Mz*_:

(27) $$\begin{array}{c} M_{z}\left(k+1\right)=M_{z}\left(k\right)+\delta_{Mz}\nabla Wt_{\mu }. \end{array}$$

Equation (28) gives the updated fuzzification membership function with centre value as *M*_*x*_ and learning rate as *δ*_*Mx*_:

(28) $$\begin{array}{c} M_{x}\left(k+1\right)=M_{x}\left(k\right)+\delta_{Mx}\nabla M_{x}. \end{array}$$

Algorithm 3 summarizes the process of the fuzzy convolutional neural network algorithm.

## 5. Experimental Evaluation

### 5.1. Dataset Descriptions

The image classification is an important task in the proposed system which classifies the image dataset into normal dataset and disease affected dataset. Here, the experiments are performed on medical datasets for evaluating the proposed algorithm using parameters like specificity, sensitivity, and accuracy. Besides, the other existing algorithms like decision tree (DT), Naïve Bayes (NB), K-nearest neighbour (KNN), and artificial neural network (ANN) are also compared with the proposed algorithm for analysing the efficiency of the proposed algorithm. For evaluating the effect of preprocessing techniques on classification of medical images, two different types of datasets acquired through noninvasive modalities like MRI are utilised. The datasets comprise BRATS images [41] and Brain MRI [42]. They are utilised for training, testing, and validation of the proposed algorithm.  Table 1 gives the description of the datasets utilised for classification.

### 5.2. Classification Results

#### 5.2.1. Parameter Settings

The parameter settings of proposed algorithm and the comparative algorithms for classification are presented in Table 2. Here, 75% datasets are utilised for training the classifier and 25% datasets are utilised for testing the classifier. The proposed approach is implemented in Matlab platform.

#### 5.2.2. Evaluation Parameters

The parameters like specificity, accuracy, sensitivity, and F-measure are considered in the evaluation process. The evaluation parameters are determined using the number of true positive (TP), number of false positive (FP), number of true negative (TN), and number of false negative (FP). The specificity, accuracy, and sensitivity are illustrated as follows:

(29) $$\begin{array}{c} \text{specificity}=\frac{\text{TN}}{\text{TN}+\text{FP}}, \end{array}$$

(30) $$\begin{array}{c} \text{sensitivity}=\frac{\text{TP}}{\text{TP}+\text{FN}}, \end{array}$$

(31) $$\begin{array}{c} \text{accuracy}=\frac{\text{TN}+\text{TP}}{\text{TN}+\text{TP}+\text{FN}+\text{FP}}. \end{array}$$

In true positive (TP), the right values will be predicted as the correct value. In true negative (TN), the right values will be predicted as the wrong value. In false positive (FP), the false values will be predicted as the right value, and in false negative (FN), the false value will be predicted as the wrong value.

**Figure 5:**
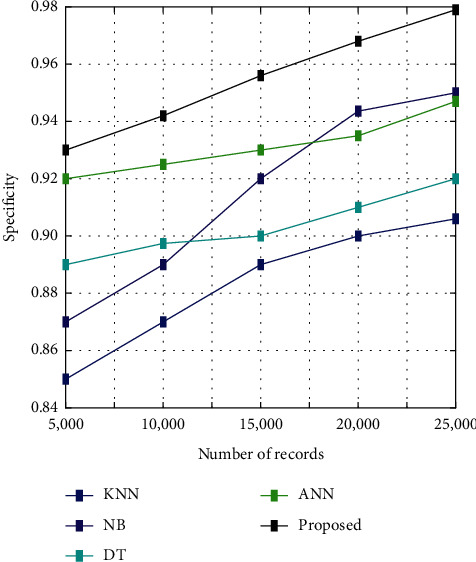
Specificity analysis.

Figure 5 illustrates the analysis of specificity among the proposed algorithm and other existing algorithms such as KNN, NB, DT, and ANN. The analysis is conducted for five different sets of images such as 5000, 10000, 15000, 20000, and 25000. It shows that the specificity is high for the proposed algorithm than the other existing algorithms like KNN, NB, DT, and ANN.

**Figure 6:**
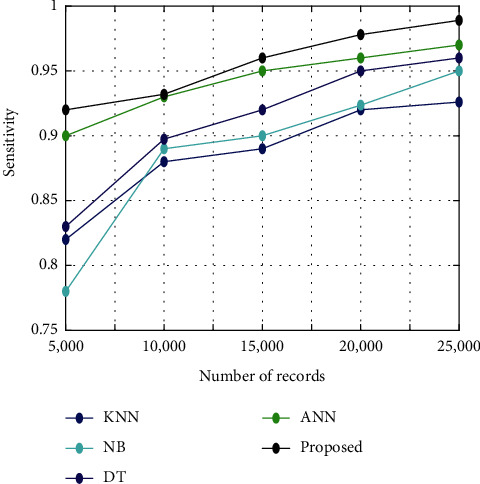
Sensitivity analysis.

Figure 6 illustrates the analysis of sensitivity among the proposed algorithm and other existing algorithms such as KNN, NB, DT, and ANN. The analysis is conducted for five different sets of images such as 5000, 10000, 15000, 20000, and 25000. It shows that the sensitivity is high for the proposed algorithm than the other existing algorithms like KNN, NB, DT, and ANN.

**Figure 7:**
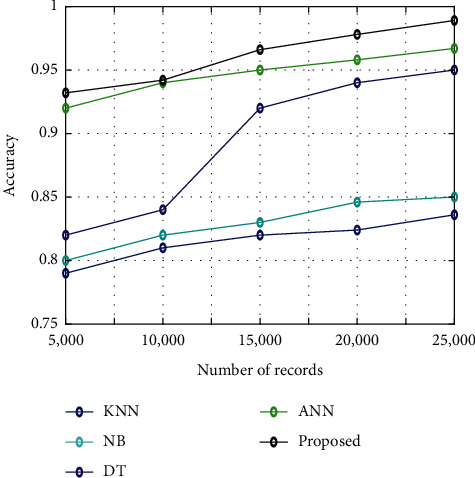
Classification accuracy.

Figure 7 illustrates the classification accuracy among the proposed algorithm and other existing algorithms such as KNN, NB, DT, and ANN. The analysis is conducted for five different sets of images such as 5000, 10000, 15000, 20000, and 25000. It shows that the accuracy is high for the proposed algorithm than the other existing algorithms like KNN, NB, DT, and ANN.

F-measure is utilised for measuring the effectiveness of the classification process. If the F-measure value is high, the predicting potential of classification process will be high. The F-measure is evaluated from the mean values of recall and precision. The recall is measured by the ratio of true positive values to the sum of true positive and false negative values. Instead, the precision is measured by the ratio of true positive values to the sum of true positive and false positive values. The F-measure based on precision and recall is expressed as

(32) $$\begin{array}{c} F-\text{measure}=2\times \;\frac{\text{precision}\times \text{recall}}{\text{precision}+\text{recall}}. \end{array}$$

**Figure 8:**
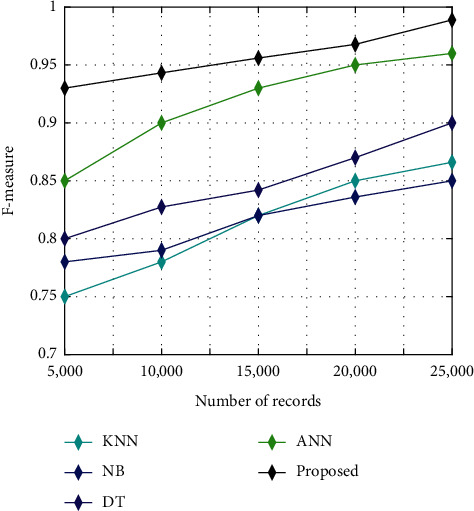
F-measure analysis.

Figure 8 illustrates the analysis of F-measure among the proposed algorithm and other existing algorithms like KNN, NB, DT, and ANN. The analysis is conducted for five different sets of images such as 5000, 10000, 15000, 20000, and 25000. It shows that the F-measure is high for the proposed algorithm than the other existing algorithms like KNN, NB, DT, and ANN.

The comparison results are given for the classification methods mentioned above. From the results, it is known that the proposed approach has superior performance over other classification methods. Particularly, the proposed approach has been able to attain better accuracy compared to other techniques like KNN, NB, DT, and ANN, respectively. In case of specificity, the proposed approach has competently acquired the higher value as compared to KNN, NB, DT, and ANN. Similarly, the proposed approach yielded better value for sensitivity and F-measure, which is comparatively higher than other classification methods. Hence, it is concluded that, for medical image classification, the proposed model is highly efficient.

#### 5.2.3. Computation Time for Classification

Table 3 displays the computation time between the proposed fuzzy convolutional neural network algorithm and other existing algorithms like KNN, NB, ANN, and DT. It displays that the computation time is greater for the proposed algorithm than the other existing algorithms like KNN, NB, DT, and ANN.

#### 5.2.4. Statistical Analysis

**Figure 9:**
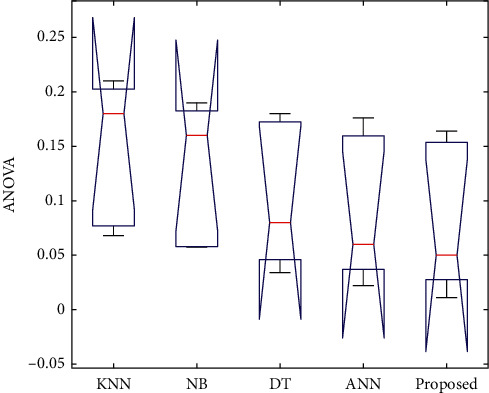
ANOVA test.

The statistical analysis was performed for analysing the improvement in the performance of the proposed algorithms than the other existing algorithms. It analyses classification problems such as the error rates and classification accuracy. Many tests were conducted for this statistical analysis which includes post hoc test, Dunnett test, Tukey test, Friedman test, and ANOVA test. For concluding whether there is any statistical difference among the proposed algorithm and other compared algorithms, a one-way analysis of variance (ANOVA) test has been conducted which analyses the statistical correctness of the proposed algorithms over other existing algorithms. It comprises mean and variance for determining the test statistic. The test statistic is then utilised for determining either the group of data is same or different. The box plots for ANOVA test are illustrated in Figure 9.

Table 4 gives the applications of CNN-based methods for medical image retrieval, computer-aided diagnosis, and classification task. Convolutional neural networks have proven to provide high performance in medical image processing than the other techniques. Thus, CNNs can be successfully applied for various tasks in medical image analysis. The results may differ based on choice of CNN model, number of classes, and number of images used.

### 5.3. Encryption Results

#### 5.3.1. Analysis of Information Entropy

Entropy is known as the significant aspect of randomness, and it can be utilised for defining the degree of uncertainty of the image. Information entropy computes the uniform distribution of gray pixel throughout the image. If the information entropy value is greater, the image confusion level of the encrypted image will be high. Assume *E* as the source of information and the information entropy of *E* is computed as follows:

(33) $$\begin{array}{c} F\left(E\right)=-\sum_{i=1}^{n}p\left(E_{i}\right)\log_{2}\frac{1}{p\left(E_{i}\right)}, \end{array}$$

where all the possibilities of *E*_*i*_ is denoted as *n* and the probability of occurrence of *E*_*i*_ is denoted as *p*(*E*_*i*_). Based on the equation, information entropy for the proposed algorithm is analysed in the encrypted image.

The comparative analysis is carried out in the proposed system for analysing the influence of proposed encryption algorithm in the security performance of medical images. Different types of encryption algorithms are utilised for comparative analysis of proposed algorithm. The most commonly used approaches for the encryption process are feed-forward, feed-forward backpropagation, and fitting neural network algorithms. All these algorithms have been proved as highly effective in many security-related approaches. The information entropy results for the proposed algorithm, and other existing algorithms are displayed in Table 5. From the table, it is observed that the information entropy value is high for the proposed encryption algorithm than the other existing algorithms.

#### 5.3.2. Correlation Coefficients

Correlation analysis is the statistical testing process which has the ability to crack the encryption algorithm. Also, the correlation coefficients provide evaluation standard for testing the encryption algorithm. For plain images, the correlation between adjacent pixels will be generally high. In order to avoid the statistical attacks due to correlation among adjacent pixels, correlated coefficients must be reduced for encrypted image. Here, the correlation coefficient of different pixels is utilised for determining the ability of encryption algorithm and to minimise the correlation between adjacent pixels. Correlation analysis of encrypted image considers all the possible adjacent situations such as diagonal, vertical, and horizontal directions. The following equations are utilised for assessing the correlation between the adjacent pixels in encrypted and plain images:

(34) $$\begin{array}{c} r_{xy}=\frac{\mathrm{cov}\left(x,y\right)}{\sqrt{A\left(x\right)A\left(y\right)}},\\ \mathrm{cov}\left(x,y\right)=\frac{1}{M}\sum_{i=1}^{M}\left[x_{i}-B\left(x\right)\right]\left[y_{i}-B\left(y\right)\right],\\ B\left(x\right)=\frac{1}{M}\sum_{i=1}^{M}x_{i},\\ A\left(x\right)=\frac{1}{M}\sum_{i=1}^{M}{\left[x_{i}-E\left(x\right)\right]}^{2}, \end{array}$$

where  *M*  is represented as the overall pixels of the image and *x* and *y* are denoted as the neighbouring pixel values. The mean values are represented as *B*(*x*) and *B*(*y*), and the covariance and variance are represented as cov(*x*, *y*) and *A*(*x*). Based on the correlation equations, 2000 sets of adjacent pixels are selected from the diagonal, vertical, and horizontal directions. During the proposed encryption process, the pixels in the image are entirely randomized so that there will be no leaking of statistical information from encrypted image. Hence, the proposed algorithm has the potential to withstand statistical attack.

**Figure 10:**
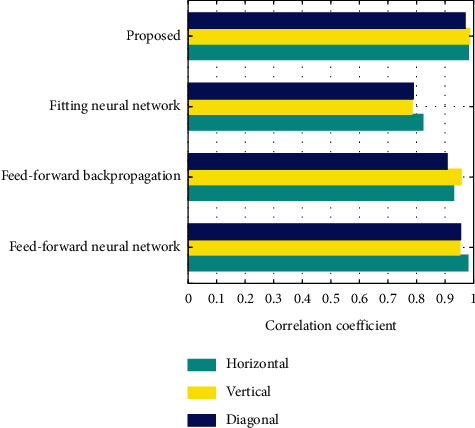
Correlation coefficient of plain image.

Figure 10 displays the correlation coefficients of plain image for the proposed encryption algorithm and other existing algorithms such as feed-forward, feed-forward backpropagation, and fitting neural network in diagonal, vertical, and horizontal directions. From the figure, it can be observed that the correlation coefficients of plain image are very high for the proposed encryption algorithm than the other existing algorithms in all directions.

**Figure 11:**
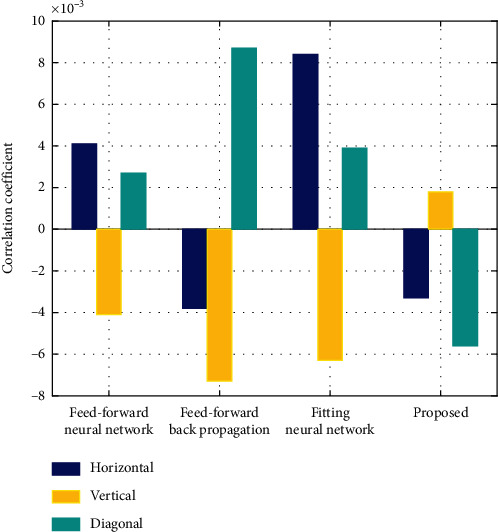
Correlation coefficient of encrypted image.

Figure 11 displays the correlation coefficient of encrypted image for the proposed algorithm and other existing algorithms such as feed-forward, feed-forward backpropagation, and fitting neural network in horizontal, vertical, and diagonal directions. From the figure it is known that the correlational coefficient of encrypted image is comparatively less for the proposed algorithm than the other existing algorithms in all directions.

#### 5.3.3. Differential Attack Analysis

Generally, a small variation in the original image can lead to noticeable variation in the encrypted image. To measure these changes in the original image, unified average changing intensity (UACI) and number of pixels change rate (NPCR) are utilised. The objective of NPCR is to calculate the exact number of altered pixel values in the differential attack. If the NPCR value is high, the result will be better. Instead, UACI focuses on the average variance between two paired encrypted images. If the value of UACI is low, the result will be better. NPCR and UACI quantitatively and qualitatively analyse the processed image and measure the sensitivity of original image. They can also determine whether the images will be able to resist differential attack. The UACI and NPCR are computed by the following equations:

(35) $$\begin{array}{c} \text{NPCR}=\frac{1}{m\times n}\left[\sum_{i.j}H\left(i,j\right)\right]\times 100\%, \end{array}$$

(36) $$\begin{array}{c} \text{UACI}=\frac{1}{m\times n}\left[\sum_{i,j}\frac{E_{1}\left(i,j\right)-E_{2}\left(i,j\right)}{225}\right]\times 100\%, \end{array}$$

where *E*_1_ and *E*_2_ are represented as an encrypted image before and after changing one pixel of the plain image. If *E*_1_(*i*, *j*) ≠ *E*_2_(*i*, *j*), then *H*(*i*, *j*)=1, if not *H*(*i*, *j*)=0. The estimated average UACI value is 33.46% and NPCR value is 99.56%. If the proposed algorithm attains this value, then the algorithm will have better performance.

The experimental results for NPCR and UACI are compared with other existing encryption algorithms such as feed-forward, feed-forward backpropagation, and fitting neural network.  Table 6 displays the results of UACI and NPCR for the proposed and other existing encryption algorithms. The values from the table illustrate that, for NPCR, the proposed algorithm has the highest value than the other existing algorithms. But for UACI, feed-forward neural network has the lowest value than the other algorithms.

#### 5.3.4. Computational Time Analysis

The time consumed by the algorithm for encryption and decryption processes is utilised for analysing the efficiency of proposed algorithm. However, the time consumed by the same algorithm will be varied for different hardware platforms.  Table 7 displays the computation time of the proposed and the other existing algorithms such as feed-forward, feed-forward backpropagation, and fitting neural network. From the table, it is observed that the computation time required for the decryption process in the proposed encryption algorithm is less when it is compared with the other existing algorithms.

## 6. Discussion

In this study, IoT  and cloud computing-based medical image analysis using fuzzy convolutional neural network has been proposed. IoT and cloud computing are the emerging technology and has been applied successfully in many fields. Many research works are conducted for utilising IoT and cloud computing in medical field. They can be effectively utilised for storing, processing, and sharing. Even though IoT and cloud computing has many advantages, it is not widely employed in the medical field due to the security aspects. *Cancer* has grown as the deadly disease for human beings from children to older generations. The earlier prediction of this disease can considerably reduce the mortality rate. By utilising IoT and cloud computing, the time required for predicting the disease can be minimised. This is because the data generated from the medical devices can be directly transferred to the cloud platform through Internet. Thus, the time required for the manual transmission of medical data will be reduced. Similarly, the image processing is carried out in the cloud, and thus, the processing time is also minimised. In medical images, the accuracy of the result should be precise for the effective treatment. Thus, an effective image processing technique is necessary for the effective prediction of diseases. Classification is one of the major tasks during image processing, and for obtaining more accurate classification results, the fuzzy logic system is integrated with convolutional neural network to form fuzzy convolutional neural network. It classifies the images into disease infected and not infected, and the results will be immediately transferred to the doctors and healthcare works. Thus, it helps the doctors to analyse the results and treat the patients at the initial stage. Furthermore, in order to increase the security and privacy of the data, encryption process based on BP neural network is employed in the proposed study. The perspective of this study is to create an efficient IoT-based cloud computing environment for earlier and accurate prediction of diseases. The proposed system can be effectively utilised in cancer centres for earlier prediction of disease and also analysing the progress in the treatment process. The proposed system can be also applied in large hospitals and healthcare centres with numerous patients' data. Implementing the IoT-based cloud computing system in the hospitals can greatly reduce the storage, and also it will ease managing patient's data.

## 7. Limitation of Proposed Approach and Future Prospects

The CNN has provided better performance for many applications in clinical domain, but it still has some limitations. More computational power and huge amount of training data are required for the architecture of CNN. If there is any deficiency in computational power, more time is required for training the data which depends on the size of the training data. These limitations can be minimised by better architecture of CNNs, increasing the number of digitally stored medical images, improved computational power, and enhanced data storage facilities. IoT-based cloud computing can be utilised for enhancing the data storage facilities. Even though the Internet of Things and cloud computing can be of huge benefit to healthcare, there are still major challenges to tackle before the complete implementation of connected devices in healthcare. Security and privacy are the major concerns which prevent the users from utilising IoT technology for medical purposes, as healthcare monitoring systems have the possibility to get hacked or breached. The disclosure of sensitive information about the patient's health and location and interfering with sensor data may cause great consequences, which would counteract the advantages of IoT. For privacy protection, encryption and decryption processes are utilised in the proposed approach. However, the encrypted image can be potentially decrypted without acquiring the key if there is an availability of considerable computational resources and skills. Thus, the skilled hackers may be able to decrypt the encrypted data. Also, failure or bugs in the hardware or even power failure can impact the performance of sensors and connected equipment placing healthcare operations at risk. In addition, skipping a scheduled software update may be even more hazardous than skipping a doctor checkup. Moreover, regarding IoT protocols and standards, there is no consent, so devices made by different manufacturers may not work well together. The lack of uniformity prevents full-scale integration of IoT, therefore limiting its potential effectiveness. Although IoT promises to reduce the cost of healthcare in the long-term, the cost of its implementation in hospitals and staff training is quite high.

## 8. Conclusion

IoT and cloud computing are considered as the significant techniques in big data processing since they are closely related to each other. The experimental process mainly focuses on utilising both IoT and cloud computing in healthcare procedure especially in brain tumour prediction. The dataset required for disease diagnosis is collected and securely transformed to the cloud. Then, the fuzzy convolutional neural network algorithm is proposed for medical image diagnosis. The proposed algorithm classifies the images into normal and abnormal images, and the results from the analysis are transferred to the doctors and healthcare providers with the help of IoT for further treatment process. From the results, it is concluded that the proposed algorithm outperforms other existing algorithms and can be effectively utilised for image diagnosis. In future, other deep learning neural network algorithms can be applied for improving the accuracy of disease prediction and for efficiently analysing large volume of medical data by utilising the medical resources.

## Figures and Tables

**Algorithm 1:**
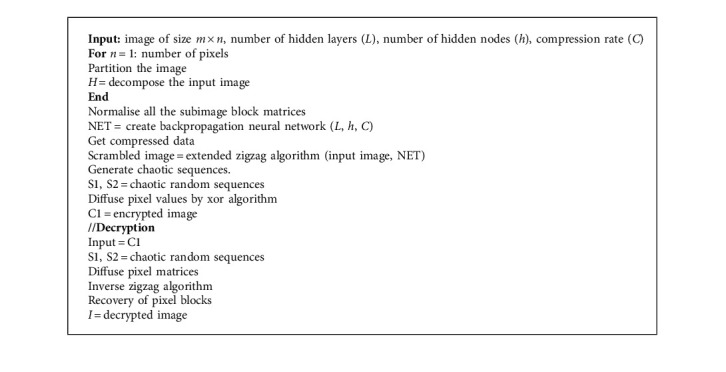
Medical image encryption and decryption.

**Algorithm 2:**
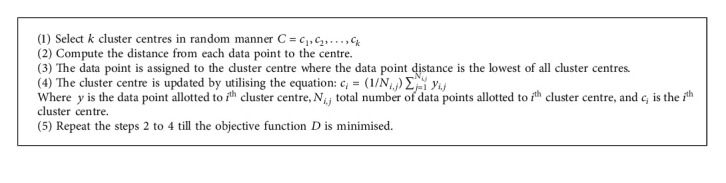
K-means clustering.

**Algorithm 3:**
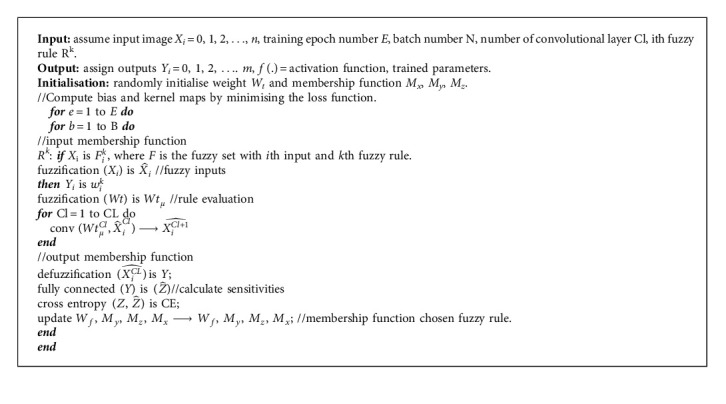
Fuzzy convolutional neural network algorithm.

**Table 1:** Dataset description.

Dataset	BRATS images	Brain cancer
Imaging modality	MRI	MRI
Total images	469	81,325
Number of patients	300	49

**Table 2:** Parameter settings.

Algorithm	Parameters	Values
KNN	Number of neighbours	5
	Distance function	Euclidean distance
	(N × D) training data	N, no. of samples; D, dimensionality of each data point
	(M × D) testing data	M, no. of data points
NB	Model	Gaussian base distribution
	N	Size of data
DT	Splitting criterion	Gini
	Minimum instances per leaf	2
ANN	Size of input layer	Size of data
	Type of ANN	Feed-forward
	Number of neurons	20
	Training and testing set	75% of training and 25% of testing set
FCNN	Input	56 × 28
	Fuzzification	2 × (input)-Gaussian MF
	In and out channel range	1 to 100
	Stride and padding	1 & 0
	Conv3x d	2 × (in & out channels, kernel size = (3 × 128), stride & padding), ReLU, Max_Pooling (55 × 1)
	Conv4x d	2 × (in & out channels, kernel size = (4 × 128), stride & padding), ReLU, Max_Pooling (54 × 1)
	Conv5x d	2 × (in & out channels, kernel size = (5 × 128), stride & padding), ReLU, Max_Pooling (53 × 1)
	Defuzzification	2 × 128

**Table 3:** Computation time.

Techniques	Time (sec)
KNN	150.012
NB	178.329
DT	140.000
ANN	60.001
Proposed	54.091

**Table 4:** Recent medical applications of CNN-based methods.

Method	Dataset	Number of images/classes	Application	Accuracy (%)
Convolutional neural network [43]	Kaggle dataset	80000 images, 5 classes	Diabetic retinopathy	75
Pretrained convolutional neural network [44]	Ultrasound images	15000 ultrasound images, 2 classes	Thyroid nodule diagnosis	83
Convolutional restricted Boltzmann machine [45]	ILD (interstitial lung diseases) CT scans	73 CT scans, 5 classes	Lung texture classification and airway detection	89
Fuzzy-based pooling in convolutional neural network [46]	MNIST dataset	60000 grayscale images, 10 classes	Image classification	94.4
	CIFAR-10 dataset	60000 RGB images, 10 classes		27.92
Tree-based convolutional neural network [19]	NWPU-VHR-10 dataset	More than 60000 images	Object classification in segmented satellite images	96.5

**Table 5:** Information entropy results.

Technique	Information entropy
Feed-forward	7.986
Feed-forward backpropagation	7.987
Fitting neural network	7.941
Proposed	7.999

**Table 6:** NPCR and UACI results.

Technique	NPCR	UACI
Feed-forward	99.412	33.615
Feed-forward backpropagation	99.702	33.572
Fitting neural network	99.684	33.551
Proposed	99.821	33.617

**Table 7:** Time taken for image encryption and decryption.

Technique	Encryption (sec)	Decryption (sec)
Feed-forward neural network	0.132	0.121
Feed-forward backpropagation	0.264	0.119
Fitting neural network	0.195	0.245
Proposed	0.174	0.109

## Data Availability

The image data used to support the findings of this study are included within the article. We can visit https://www.smir.ch and create an account to get the BRATS dataset.
